# Intranasal Immunization with DOTAP Cationic Liposomes Combined with DC-Cholesterol Induces Potent Antigen-Specific Mucosal and Systemic Immune Responses in Mice

**DOI:** 10.1371/journal.pone.0139785

**Published:** 2015-10-06

**Authors:** Rui Tada, Akira Hidaka, Naoko Iwase, Saeko Takahashi, Yuki Yamakita, Tomoko Iwata, Shoko Muto, Emi Sato, Noriko Takayama, Emi Honjo, Hiroshi Kiyono, Jun Kunisawa, Yukihiko Aramaki

**Affiliations:** 1 Department of Drug Delivery and Molecular Biopharmaceutics, School of Pharmacy, Tokyo University of Pharmacy and Life Sciences, Tokyo, Japan; 2 Division of Mucosal Immunology and International Research and Development Center for Mucosal Vaccines, Department of Microbiology and Immunology, The Institute of Medical Science, The University of Tokyo, Tokyo, Japan; 3 Laboratory of Vaccine Materials, National Institute of Biomedical Innovation, Osaka, Japan; The Ohio State University, UNITED STATES

## Abstract

Despite the progress made by modern medicine, infectious diseases remain one of the most important threats to human health. Vaccination against pathogens is one of the primary methods used to prevent and treat infectious diseases that cause illness and death. Vaccines administered by the mucosal route are potentially a promising strategy to combat infectious diseases since mucosal surfaces are a major route of entry for most pathogens. However, this route of vaccination is not widely used in the clinic due to the lack of a safe and effective mucosal adjuvant. Therefore, the development of safe and effective mucosal adjuvants is key to preventing infectious diseases by enabling the use of mucosal vaccines in the clinic. In this study, we show that intranasal administration of a cationic liposome composed of 1,2-dioleoyl-3-trimethylammonium-propane (DOTAP) and 3β-[N-(N',N'-dimethylaminoethane)-carbamoyl] (DC-chol) (DOTAP/DC-chol liposome) has a potent mucosal adjuvant effect in mice. Intranasal vaccination with ovalbumin (OVA) in combination with DOTAP/DC-chol liposomes induced the production of OVA-specific IgA in nasal tissues and increased serum IgG1 levels, suggesting that the cationic DOTAP/DC-chol liposome leads to the induction of a Th2 immune response. Additionally, nasal-associated lymphoid tissue and splenocytes from mice treated with OVA plus DOTAP/DC-chol liposome showed high levels of IL–4 expression. DOTAP/DC-chol liposomes also enhanced OVA uptake by CD11c^+^ dendritic cells in nasal-associated lymphoid tissue. These data demonstrate that DOTAP/DC-chol liposomes elicit immune responses via an antigen-specific Th2 reaction. These results suggest that cationic liposomes merit further development as a mucosal adjuvant for vaccination against infectious diseases.

## Introduction

Globally, infectious diseases are still one of the most important risk factors for human disease and the second leading cause of death [1, 2]. Despite the progress modern medicine has made to date, successful prevention and control of life-threatening infections remain a significant challenge. In the past two decades, there has been an increase in the number of infectious diseases worldwide due to the increased use of immunosuppressive therapies and the emergence of antibiotic-resistant microbes [3]. Therefore, there is a great need for the development of novel antimicrobial agents or anti-infective strategies.

Vaccination is a key approach to preventing illness and death caused by infectious disease. Mucosal vaccines are a promising strategy for preventing infectious diseases since mucosal surfaces are a major route of entry for most pathogens and mucosal adjuvants are known to induce potent systemic and mucosal antigen-specific immune responses [4–6]. Recent vaccine research has focused on the production of antibodies at mucosal sites to prevent pathogen entry into the host [7–9]. However, such approaches have proven impractical for clinical use due to safety and efficacy concerns. The majority of approved vaccines worldwide are administered by subcutaneous or intramuscular injection and induce systemic immune responses but not mucosal immune responses. To solve this problem, the development of mucosal vaccines is essential. To attain that goal, an appropriate mucosal adjuvant is needed because of the inherently poor immunogenicity of protein antigens when administered by the mucosal route [10]. Recently, intranasal injection of pathogenic microbe-derived antigens combined with a potent mucosal adjuvant was shown to be effective against infections such as influenza [11]. The advantages of intranasal administration are as follows: (a) it is a non-invasive (and painless) route of antigen delivery, resulting in improved patient compliance, and (b) rapid absorption into systemic circulation via the epithelial layer allows induction of a systemic effect [12–14]. However, the agents used as adjuvants, such as cholera toxin [15] and heat-labile enterotoxin [16], which are produced by pathogenic strains of *Vibrio cholera* and *Escherichia coli*, respectively, can have unexpected adverse effects due to their toxicity and antigenicity. Therefore, the development of safe and effective mucosal adjuvants is key to preventing infectious diseases by enabling the use of mucosal vaccines in the clinic.

Recent advances have shown that cationic substances and nanoparticles possess adjuvant properties when combined with antigens in vaccines [17–20]. Our laboratory has carried out extensive studies on liposomes with respect to their effects on immunological responses [21–25]. Liposomes are lipid-based membranous vesicles used for drug delivery [26] and can serve as immunomodulators [27–29]. Cationic liposomes deliver molecules (including nucleic acids [30–33], and proteins [34]) into various cells such as antigen-presenting cells (APCs), therefore we hypothesized that they may have potential use as safe and effective mucosal adjuvants. We evaluated intranasal administration of liposomes composed of cationic lipids combined with ovalbumin (OVA) as a model antigen to determine if this combination could induce antigen-specific mucosal and systemic humoral immune responses in mice. We also evaluated the T helper (Th) response, in terms of IgG1/IgG2a balance and the expression of Th1/Th2 cytokines, in order to further characterize the mucosal adjuvant effects of cationic liposomes. Additionally, we examined whether cationic liposomes enhanced antigen uptake by dendritic cells (DCs) in nasal-associated lymphoid tissues (NALTs), in order to better understand the underlying mechanisms of the mucosal adjuvant effects of cationic liposomes.

## Materials and Methods

### Ethics statement

All animal experiments were performed in accordance with the guidelines for laboratory animal experiments of the Tokyo University of Pharmacy and Life Sciences. The institution’s committee for laboratory animal experiments approved each experimental protocol (P13-22 and P14-31).

### Animals and materials

Female BALB/c mice (6 weeks old) were purchased from Japan SLC (Shizuoka, Japan) and housed in a specific pathogen-free environment. 1,2-Dioleoyl-3-trimethylammonium-propane (DOTAP), 3β-[N-(N',N'-dimethylaminoethane)-carbamoyl] (DC-chol), L-α-phosphatidylserine from porcine brains (PS), and L-α-phosphatidylcholine from chicken eggs (PC) were purchased from Avanti Polar Lipids (Alabaster, AL, USA). Cholesterol (Chol) was obtained from Sigma-Aldrich (St. Louis, MO, USA) and cholera toxin (CT) was purchased from Wako Pure Chemical Industries (Osaka, Japan). Egg white ovalbumin (OVA) was obtained from Sigma-Aldrich, and endotoxin contaminants were removed by a phase separation technique as described previously [35].

### Preparation of liposomes

Liposomes were prepared as described previously [36]. Briefly, 10 μmol of total lipid (DOTAP:DC-chol at 1:1, DOTAP:chol at 1:1, PS: PC:chol at 2:1:1, or PC:chol at 3:1 mol ratios) was evaporated to dryness in a glass tube and desiccated for at least 1 h *in vacuo*. The lipid films that were generated were then hydrated by the addition of 250 μl of phosphate-buffered saline (PBS) and vortexed at room temperature for 5 min. The multilamellar vesicles were extruded 10 times by passage through a polycarbonate membrane with appropriate pore size (ADVANTEC, Tokyo, Japan) and sterilized by filtration through 0.45-μm filter membranes. The particle size and zeta (ζ)-potential of the liposomes were measured by NICOMP 380 ZLS (Particle Sizing Systems, Port Richey, FL, USA).

### Immunization and sampling schedule

Mice were divided into four treatment groups and immunized intranasally as follows: 1) PBS, 2) liposomes alone, 3) OVA alone, or 4) OVA in combination with liposomes (unless otherwise indicated, we used liposomes with 100 μm diameter). Each group of mice was immunized once weekly. To monitor the induction of serum antigen-specific IgG, blood samples were collected at various time points via the tail vein. The blood was allowed to clot at 25°C for 30 min, followed by incubation at 4°C for 60 min, and then serum was separated by centrifugation at 1200 *g* for 30 min and stored at -80°C until analysis by ELISA. To monitor the induction of antigen-specific IgA in nasal washes, nasal wash samples were collected immediately after the mice were sacrificed by cervical dislocation, as previously described [17].

### ELISA for detecting anti-OVA antibody in serum and nasal wash

A 96-well Nunc MaxiSorp plate (Thermo Scientific, Waltham, MA, USA) was coated with 1.25 μg OVA dissolved in 0.1 M carbonate buffer (pH 9.5) and was incubated overnight at 4°C. The plate was then washed with PBS containing 0.05% Tween 20 (PBST) and blocked with 1% bovine serum albumin (BSA; Roche Applied Science, Penzberg, Germany) containing PBST (BPBST) at 37°C for 60 min. The plate was washed and incubated with serum samples for 60 min at 37°C. For detection of anti-OVA IgG antibody, plates were washed with PBST, treated with peroxidase-conjugated anti-mouse IgG secondary antibody (Sigma-Aldrich) in BPBST, and developed using a tetramethylbenzidine (TMB) substrate system (KPL, Gaithersburg, MD, USA). For the detection of other antibody isotypes, plates were washed with PBST, treated with biotin-conjugated anti-mouse IgA, IgG1, or IgG2a secondary antibodies (BioLegend, San Diego, CA, USA) in BPBST, followed by addition of avidin-HRP (BioLegend) in PBST to each well. Plates were developed using a TMB substrate system (KPL). Colour development was terminated using 1N phosphoric acid, and the optical density was measured at 450 nm (reference filter, 650 nm). The endpoint titres were expressed as the reciprocal of the last dilution, reaching a cut-off value set to two times the mean optical density of a negative control sample [37, 38].

### Splenocyte preparation for cell culture

Splenocytes were prepared as described previously [39–41]. Briefly, immediately following sacrifice of the mice by cervical dislocation, spleens from BALB/c female mice were excised, teased apart in RPMI 1640 medium (Wako Pure Chemical Industries), and centrifuged. The single-cell suspension obtained was treated with ACK lysis buffer to lyse the red blood cells. After centrifugation, splenocytes were maintained in RPMI 1640 medium supplemented with 10% heat-inactivated fetal bovine serum (FBS; Biowest, Nuaillé, France), 100 μg/mL of streptomycin sulphate salt (Sigma-Aldrich), and 100 U/mL of penicillin G potassium salt (Sigma-Aldrich). The cells were cultured at a density of 2 × 10^6^ cells/well in a media volume of 0.5 mL in 48-well flat-bottom plates (IWAKI, Tokyo, Japan) and re-stimulated with OVA for the indicated time at 37°C in 5% CO_2_.

### Cytokine assay

Cytokine concentrations in the samples were determined using ELISA MAX^TM^ Standard Sets (BioLegend) according to the manufacturer’s instructions. The data are expressed as mean ± standard deviation for the samples, which were assayed in triplicate. At least 3 independent experiments were conducted.

### RNA extraction and reverse transcription-polymerase chain reaction (RT-PCR)

Semi-quantitative RT-PCR was performed as follows [42]: Total RNA from spleen and NALTs was extracted with the RNAiso Plus reagent (TAKARA BIO Inc, Tokyo, Japan) and quantified by spectrophotometric measurements. cDNA was synthesized from 1 μg of total RNA using ReverTra Ace (TOYOBO, Tokyo, Japan) according to the manufacturer’s instructions. cDNA was amplified with primers specific for interferon-γ (IFN-γ), interleukin–4 (IL–4), tumour necrosis factor-α (TNF-α), and β-actin, as an internal standard. The primers used for PCR were as follows: IFN-γ, forward, 5′-AGGCCATCAGCAACAACATAAG–3′ and reverse, 5′-TAGACATCTCCTCCCATCAGC–3′; IL–4, forward, 5′-GTTGTCATCCTGCTCTTCTTTCTCG–3′ and reverse, 5′-GGACTTGGACTCATTCATGGTGC–3′; TNF-α, forward, 5′-TGCCTATGTCTCAGCCTCTTCTC–3′ and reverse, 5′-CCTATGTCTCAGCCTCTCCACTTGGTGGTTTGCTACG–3′; β-actin, forward, 5′-GCACCACACCTTCTACAATGAG–3′ and reverse, 5′-GCACCACACCTTCTACAATGAG–3′. PCR was performed using appropriate conditions that were determined in preliminary experiments, and the PCR products were analysed on 2% agarose gels stained and visualized with GelRed nucleic acid gel stain (Wako Pure Chemical Industries).

### Flow cytometric analysis of antigen uptake

Alexa Fluor 488-OVA uptake was analysed with single cell suspensions isolated from NALTs of BALB/c female mice 1 h after intranasal administration of Alexa Fluor 488-OVA (5 μg/mouse; Life Technologies, Carlsbad, CA, USA) plus DiI-labelled DOTAP/DC-chol liposomes (0.4 μmol/mouse). Cells were then blocked with rat anti-mouse CD16/CD32 (Fc block; BD Biosciences, San Jose, CA, USA) and stained with PE anti-mouse CD11c (BD Biosciences) or the respective isotype control. The samples were analysed using a FACS Canto instrument (BD Biosciences).

### Fluorescence microscopy analysis of antigen localization in NALTs

To determine whether the cationic liposomes promoted antigen uptake by DCs in NALTs, BALB/c female mice were administered Alexa Fluor 488-OVA (5 μg/mouse; Life technologies) intranasally in combination with DiI-labelled DOTAP/DC-chol liposomes or DOTAP/chol liposomes (0.4 μmol/mouse). One hour after administration, the mice were euthanized and samples of NALT were excised and frozen in O.C.T. (Sakura Finetek Japan, Tokyo, Japan) on dry ice. Cryosections (8 μm thick) were cut, fixed in 4% paraformaldehyde (PFA) for 10 min at room temperature, washed with PBS, and incubated in blocking buffer for 30 min. After blocking, the sections were stained with anti-CD11c antibody (BD Biosciences) and examined with a BZ–8100 fluorescent microscope (KEYENCE, Tokyo, Japan).

### Statistical analysis

Statistical differences were assessed using the Kruskal–Wallis with Dunn’s post–hoc test and *t*-test with Welch correction for antibody and cytokine production, respectively. A two-way ANOVA with Bonferroni post–hoc test was used for body weight loss. *P* values less than 0.05 were considered significant.

## Results

### Physicochemical properties of the liposomes

We first evaluated the particle size and ζ–potential of the liposomes prepared in this study. The particle size and ζ–potential of the liposomes are listed in Table 1. We also examined the stability of cationic liposomes composed of DOTAP and DC-cholesterol (DOTAP/DC-chol liposomes) as well as DOTAP and cholesterol (DOTAP/chol liposomes). The results demonstrated that the DOTAP/DC-chol liposome was exceptionally stable compared to the DOTAP/chol liposome, as seen in S1 Fig.

**Table 1:** Physicochemical Properties of the Liposomes used in this Study.

Liposomes	Particle size (nm)	ζ potential (mV)
DOTAP/DC-chol (50 nm)	57.2 ± 1.9	9.32
DOTAP/DC-chol (100 nm)	88.6 ± 2.5	11.82
DOTAP/DC-chol (1000 nm)	846.2 ± 10.5	11.18
DOTAP/chol	86.2 ± 28.7	6.49
PS	106.4 ± 18.5	-6.05
PC	121.9 ± 9.2	-0.14

### Induction of antigen-specific serum IgG and nasal tissue IgA after administration of OVA with the cationic liposomes

**Fig 1:**
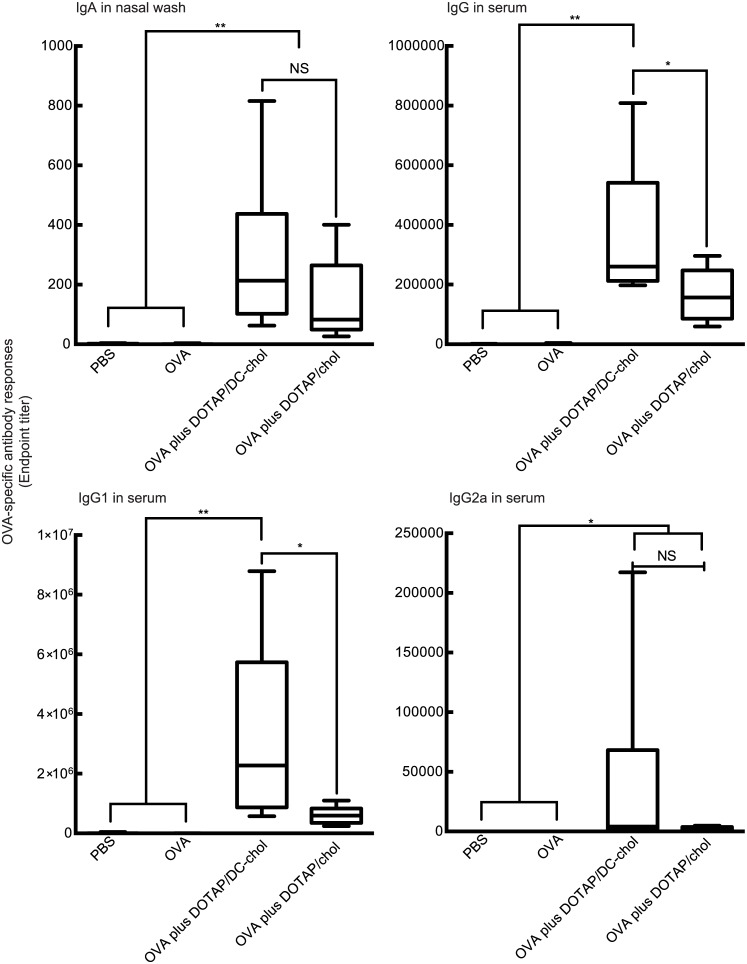
Induction of OVA-specific serum IgG and nasal tissue IgA responses in BALB/c mice immunized intranasally with OVA and cationic liposomes. BALB/c female mice were immunized intranasally with PBS, OVA (5 μg/mouse) alone, or OVA (5 μg/mouse) plus various cationic liposomes (0.4 μmol/mouse) on days 0 and 7. Serum and nasal washes were collected on day 14. The anti-OVA IgG, IgG1, and IgG2a levels in serum and anti-OVA IgA level in nasal washes were detected by ELISA assay as described in the “Materials and Methods” section. The data were obtained from at least three independent experiments. The box-plot shows the median value with the 25th-75th percentiles and the error bars indicate the 5th-95th percentiles. Significance was assessed using the Kruskal–Wallis with Dunn’s post–hoc test: *p < 0.05, **p < 0.01, NS: not significant.

We examined whether intranasal administration of the cationic liposomes acts as a mucosal adjuvant in BALB/c female mice. Mice were immunized intranasally twice weekly with OVA alone or combined with the cationic liposomes (DOTAP/DC-chol or DOTAP/chol liposomes). Intranasal vaccination with OVA and the cationic liposomes induced expression of OVA-specific IgA in nasal tissue and IgG in serum. Intranasal immunization with PBS or OVA alone did not induce OVA-specific immunoglobulin expression in nasal tissue or serum in this experiment (Fig 1).

DOTAP/DC-chol liposomes induced potent antigen-specific IgG serum responses that were superior to DOTAP/chol liposomes. DOTAP/DC-chol liposomes also induced significant antigen-specific IgG production in serum when compared to the other liposomes used in this study. In addition, DOTAP/DC-chol liposomes showed higher levels of IgA expression (median value: 213.0) in nasal washes than DOTAP/chol liposomes (median value: 83.6), but the differences were not significant (Fig 1). Taken together, DOTAP/DC-chol liposomes demonstrated potent mucosal adjuvant activity.

Since murine serum IgG subclasses have been used to assess the type of immune responses induced by immunization, we investigated the production of serum IgG1 and IgG2a after intranasal immunization with OVA and the cationic liposomes. The results demonstrated that an IgG1 response rather than IgG2a was elicited by intranasal treatment with OVA and the cationic liposomes (Fig 1), indicating that the cationic liposomes induced a Th2-biased immune response.

**Fig 2:**
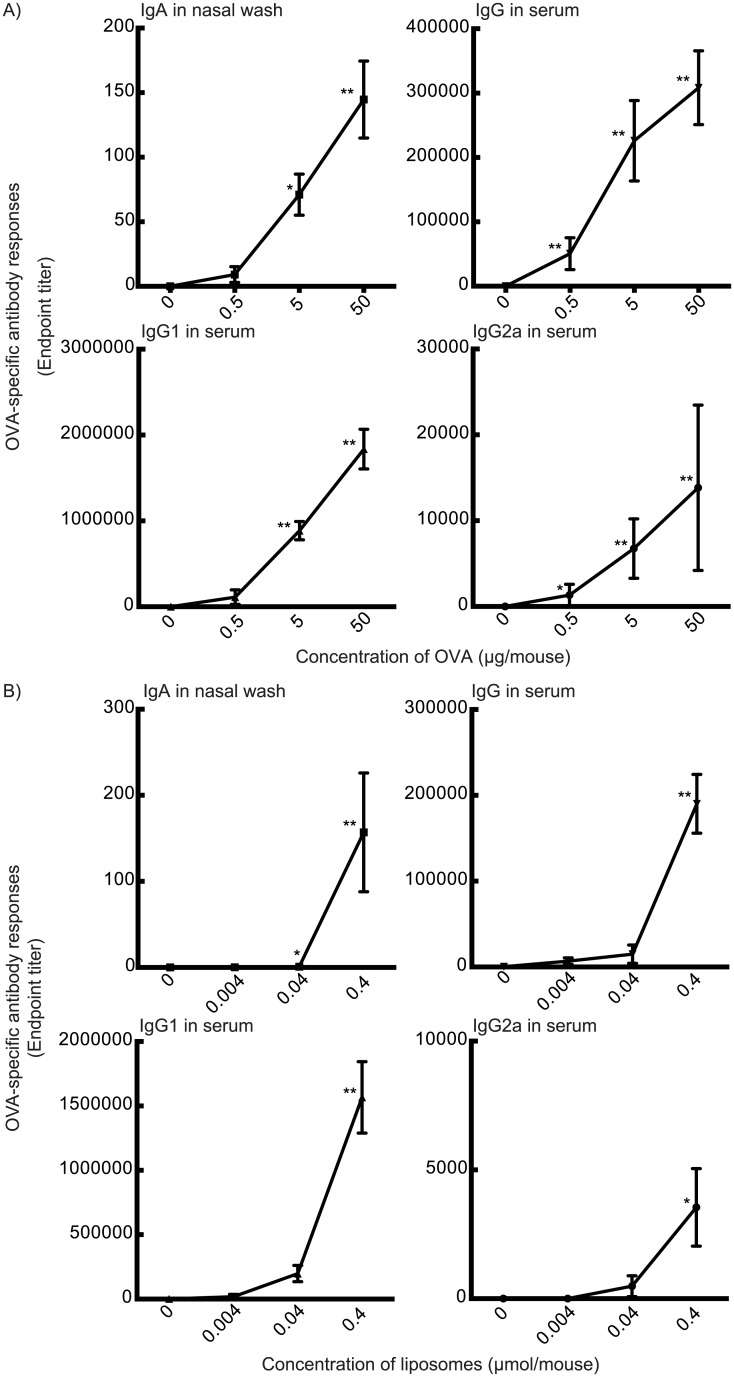
Cationic DOTAP/DC-chol liposome elicits dose-dependent OVA-specific antibody production depending on antigen (A) and liposome (B) concentrations. BALB/c female mice were immunized intranasally with various doses of OVA plus DOTAP/DC-chol liposomes on days 0 and 7. Serum was collected on day 14. The anti-OVA IgG, IgG1, and IgG2a levels in serum and anti-OVA IgA level in nasal washes were detected by ELISA assay. The data are obtained from at least three independent experiments and are expressed as the mean ± the standard error. Significance was assessed with the Kruskal–Wallis with Dunn’s post–hoc test: *p<0.05, **p<0.01.

**Fig 3:**
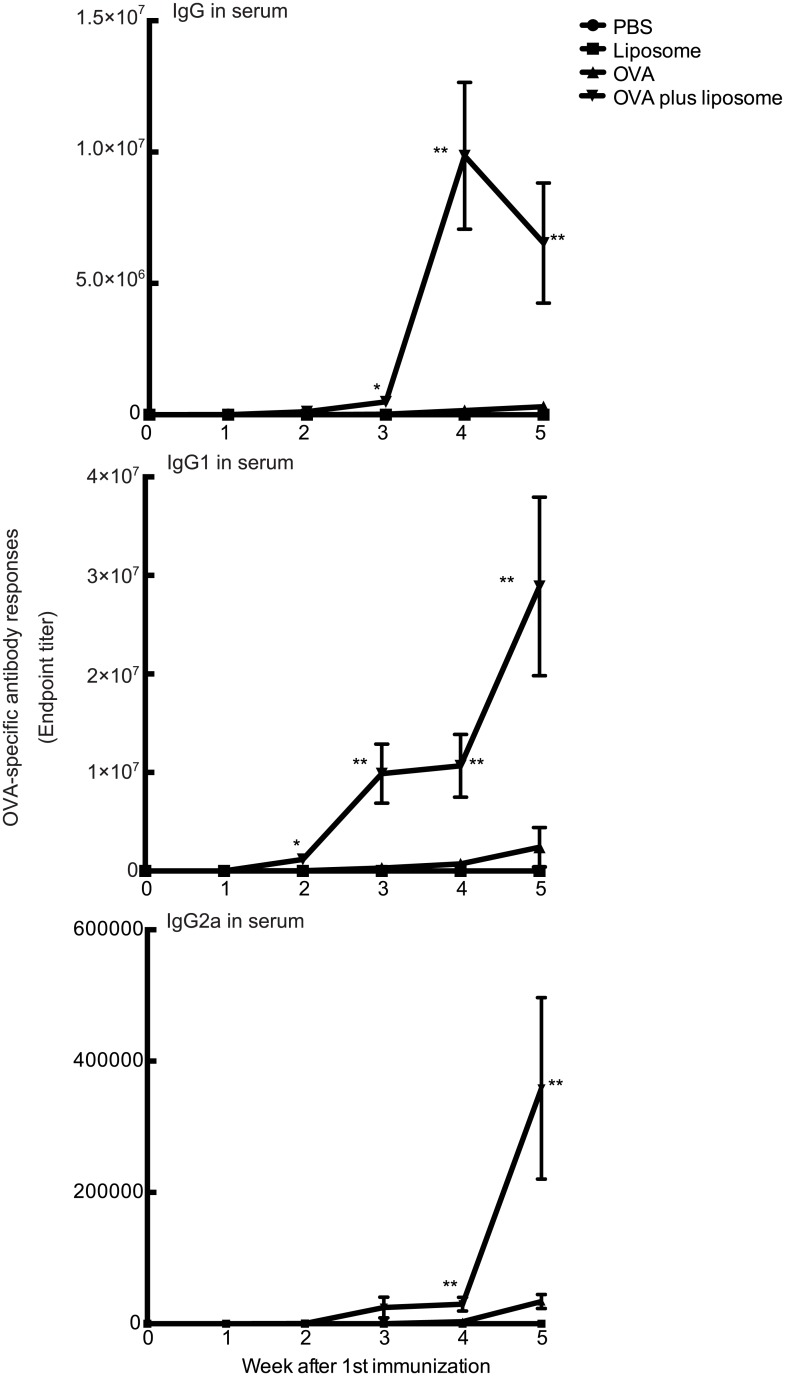
Kinetics of the appearance of OVA-specific serum IgG in BALB/c female mice immunized intranasally with OVA and DOTAP/DC-chol liposomes. BALB/c female mice were immunized intranasally with PBS alone, DOTAP/DC-chol liposomes (0.4 μmol/mouse) alone, OVA (5 μg/mouse) alone, or OVA (5 μg/mouse) plus DOTAP/DC-chol liposomes (0.4 μmol/mouse) once weekly (days 0, 7, 14, 21, and 28). Serum was collected every week immediately prior to immunization (days 0, 7, 14, 21, 28, and 35). Anti-OVA IgG, IgG1, and IgG2a levels in serum were determined by ELISA assay. The data are obtained from at least three independent experiments and are expressed as the mean ± the standard error. Significance was assessed with the Kruskal–Wallis with Dunn’s post–hoc test: *p<0.05, **p<0.0001.

We also evaluated the effect of various concentrations of the antigen and liposome on the production of nasal tissue IgA and serum IgG using DOTAP/DC-chol liposomes, which possess a higher mucosal adjuvant effect (Fig 1). Fig 2 shows that OVA-specific antibody responses were augmented in a dose-dependent manner for both antigen (Fig 2A) and liposome (Fig 2B) concentrations. The kinetics of antigen-specific IgG production was evaluated in sera collected at different time points from mice immunized once weekly (Fig 3).

On day 14 post the first immunization, OVA-specific serum IgG, IgG1, and IgG2a were detectable in sera from OVA plus DOTAP/DC-chol liposome-immunized mice, with increasing titres observed at later time points. In contrast, OVA-specific serum IgG, IgG1, and IgG2a levels from mice immunized with OVA alone demonstrated a weak level of expression on day 21- post immunization. These results show that intranasal administration of DOTAP/DC-chol liposomes markedly enhance antigen-specific mucosal and systemic immune responses in mice.

### Influence of particle size of DOTAP/DC-chol liposomes on mucosal adjuvant activity

**Fig 4:**
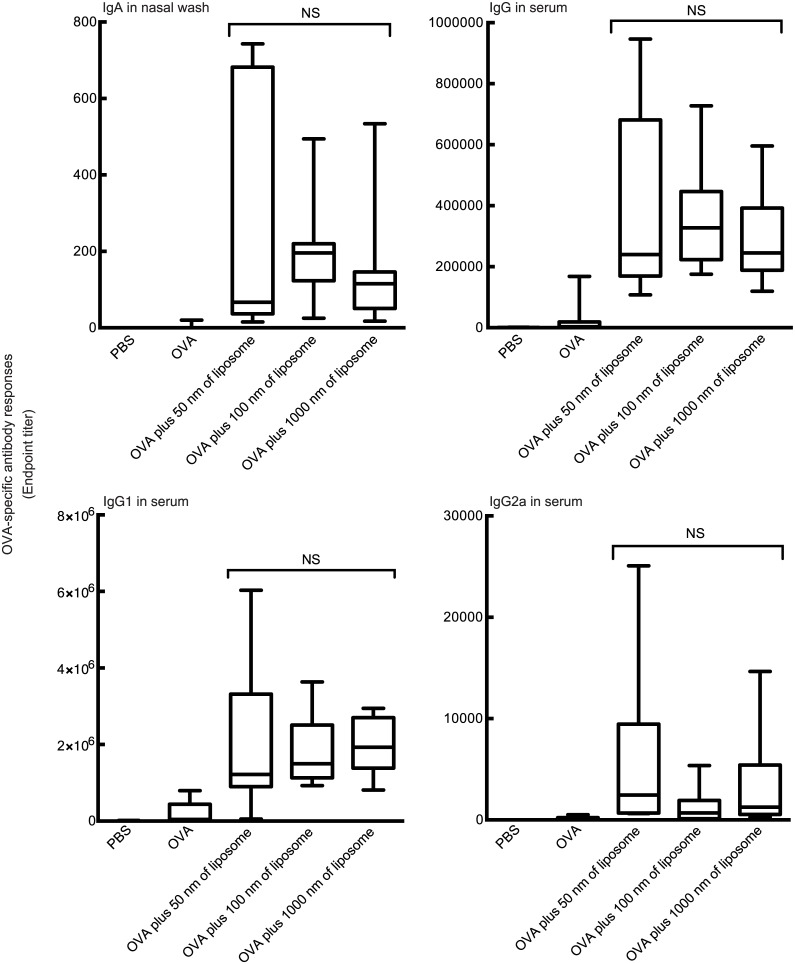
Influence of DOTAP/DC-chol liposome particle size on the mucosal adjuvant effect. BALB/c female mice were immunized intranasally with PBS, and treated with DOTAP/DC-chol liposomes of various particle sizes (0.4 μmol/mouse) alone, OVA (5 μg/mouse) alone, or OVA (5 μg/mouse) plus DOTAP/DC-chol liposomes of various particle sizes (0.4 μmol/mouse) on days 0 and 7. Serum and nasal washes were collected on day 14. The anti-OVA IgG, IgG1, and IgG2a levels in serum and anti-OVA IgA level in nasal washes were detected by ELISA assay. The data are obtained from at least three independent experiments. The box-plot shows the median value with the 25th-75th percentiles and the error bars indicate the 5th-95th percentiles. NS: not significant as assessed by the Kruskal–Wallis with Dunn’s post–hoc test.

Particle size is one of the key factors related to the biological effects of nanoparticles [43–45]. To investigate the influence of DOTAP/DC-chol liposome size on mucosal adjuvant activity, we prepared liposomes of various sizes and evaluated their mucosal adjuvant activity. The results indicated that changes in particle size did not affect DOTAP/DC-chol liposome mucosal adjuvant activity (Fig 4).

### Comparison of the mucosal adjuvant activity of DOTAP/DC-chol liposomes with a well-known potent mucosal adjuvant, CT

**Fig 5:**
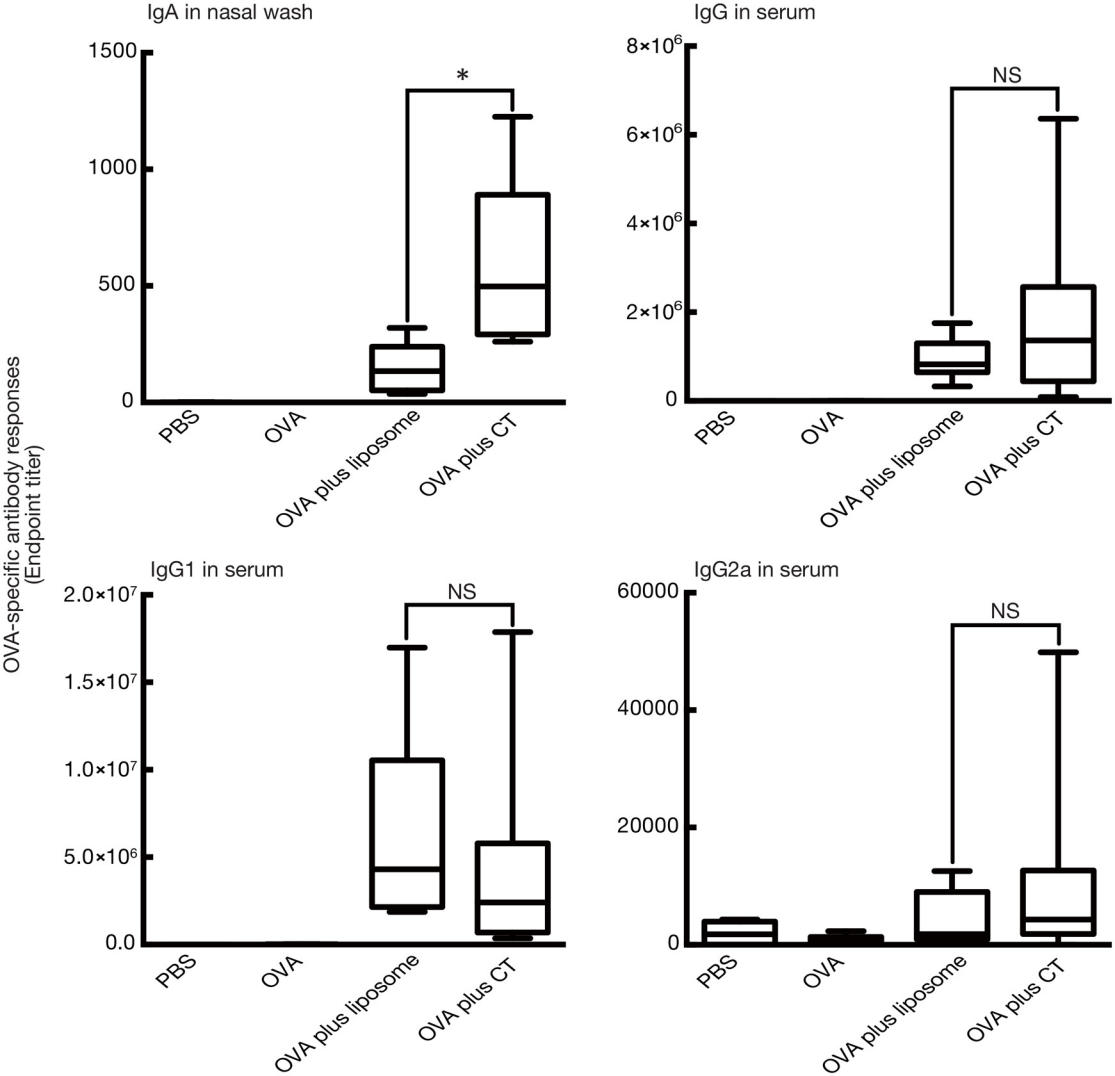
Comparison of the mucosal adjuvant activity of DOTAP/DC-chol liposomes with CT. BALB/c female mice were immunized intranasally with PBS, OVA (5 μg/mouse) alone, OVA (5 μg/mouse) plus DOTAP/DC-chol liposomes (0.4 μmol/mouse), or OVA (5 μg/mouse) plus CT (1 μg/mouse) on days 0 and 7. Serum and nasal washes were collected on day 14. The anti-OVA IgG, IgG1, and IgG2a levels in serum and anti-OVA IgA level in nasal washes were detected by ELISA assay as described in the “Materials and Methods” section. The data were obtained from at least three independent experiments. The box-plot shows the median value with the 25th-75th percentiles and the error bars indicate the 5th-95th percentiles. Significance was assessed using the Kruskal–Wallis with Dunn’s post–hoc test: *p < 0.05, NS: not significant.

Currently, experimental mucosal adjuvants are almost all pathogenic microbe-derived toxins, such as CT. Therefore, we next examined the mucosal adjuvant effect of DOTAP/DC-chol liposomes with CT, which resulted in comparable serum IgG titres (Fig 5). In addition, the production of IgA in the nasal area induced by DOTAP/DC-chol liposomes was lower compared to that induced by CT (median value: 135.1 vs 497.3).

### *In vivo* toxicity study of the intranasal administration of DOTAP/DC-chol liposomes

**Fig 6:**
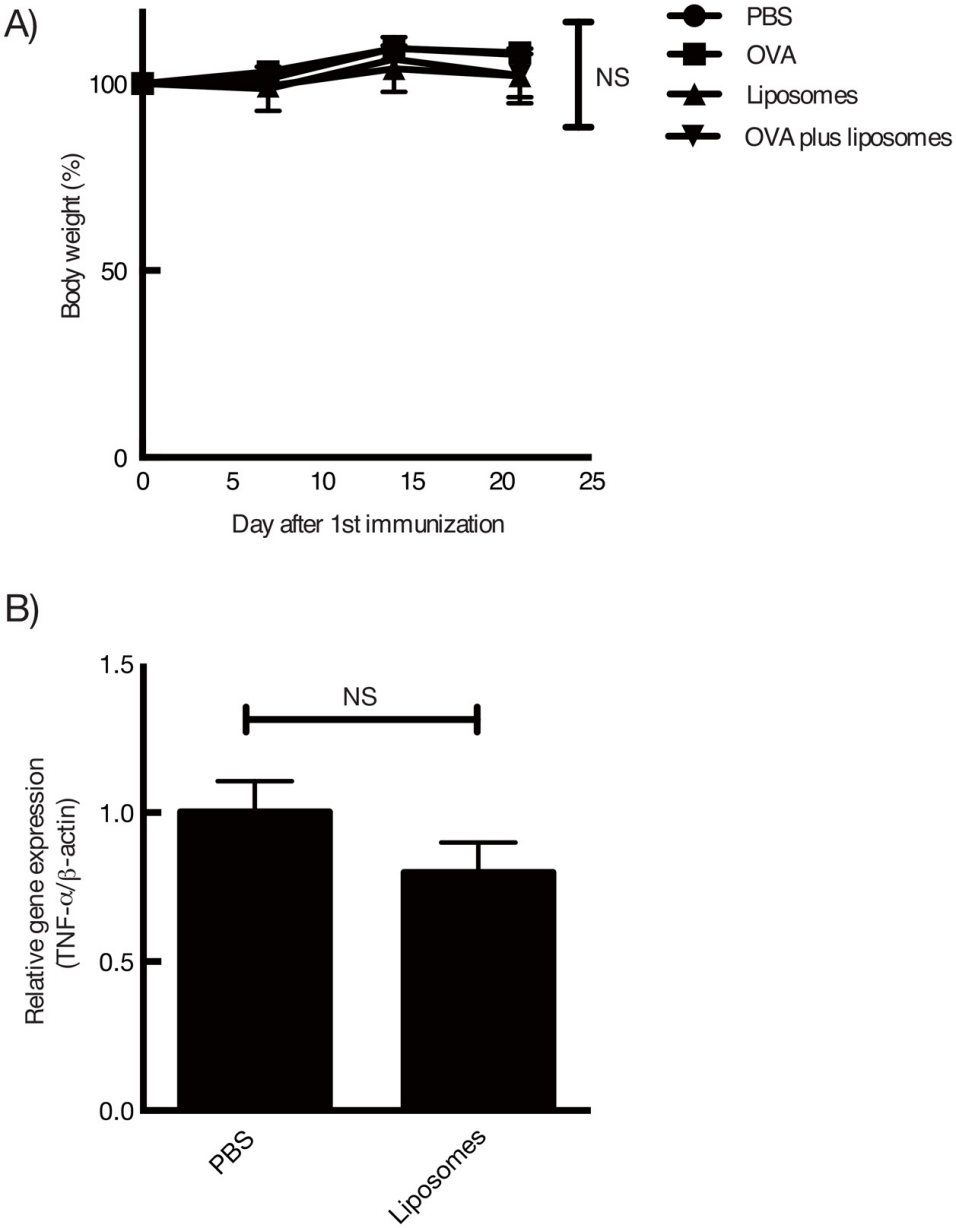
Assessment of the *in vivo* safety of DOTAP/DC-chol liposomes in mice by body weight loss and gene expression of an inflammatory cytokine, TNF-α, at the site of delivery. (A) BALB/c female mice were immunized intranasally with PBS, DOTAP/DC-chol liposomes (0.4 μmol/mouse) alone, or OVA (5 μg/mouse) plus DOTAP/DC-chol liposomes (0.4 μmol/mouse) once per week. Body weight was recorded over 21 days. Significance was assessed using a two-way repeated measures ANOVA with Bonferroni post–hoc test: NS: not significant, (B) Sixteen hours after the immunization, nasal tissues were collected and the expression of TNF-α mRNA was quantified by semi-quantitative RT-PCR. Densitometry values for mRNA were normalized to β-actin as an internal standard. The data are expressed as the mean ± standard deviation for samples assayed in triplicate. Significance was assessed using *t*-test with Welch correction: NS: not significant.

Since it has been reported that cationic liposomes show toxicity in mice [46, 47], we next examined the safety of DOTAP/DC-chol liposomes *in vivo*. To evaluate the toxicity induced by intranasal administration of DOTAP/DC-chol liposomes, we performed the following two experiments: 1) body weight loss during the course of the immunization process and 2) gene expression of an inflammatory cytokine, TNF-α, in the nasal area 16 hours after liposome administration. There were no changes in weight loss observed in the mice immunized with cationic liposomes compared to the control group (Fig 6A). Moreover, the intranasal administration of DOTAP/DC-chol liposomes did not exert TNF-α expression in the nasal area (Fig 6B). These data clearly demonstrate that, in these experimental conditions, the cationic liposomes do not result in toxicity in mice. Together with the comparison with the mucosal adjuvant CT, DOTAP/DC-chol liposomes possess the characteristics of a safe and effective mucosal adjuvant in mice.

### Effect of liposome surface charge on mucosal adjuvant activity

**Fig 7:**
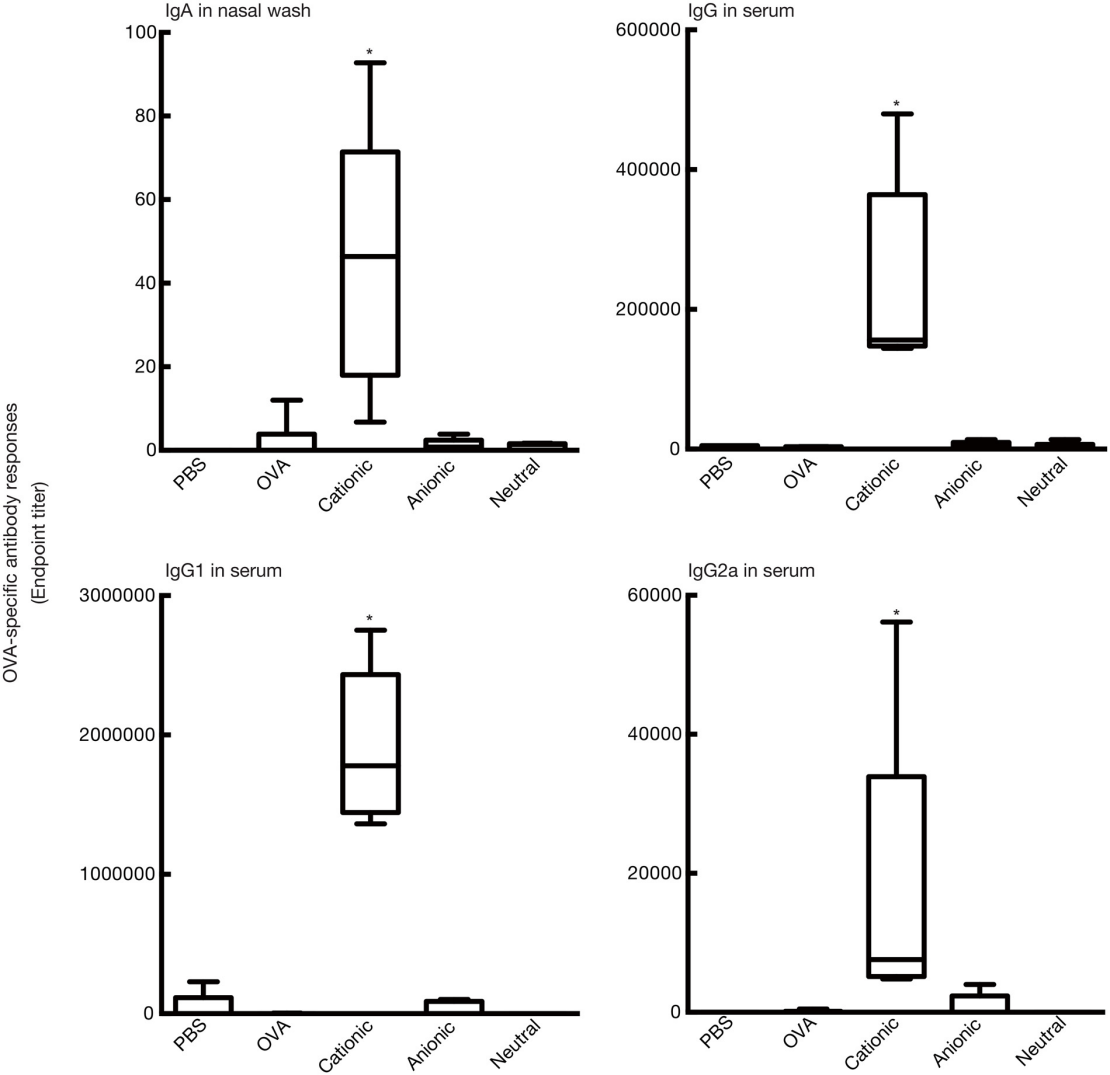
Role of liposome surface charge on the mucosal adjuvant effect. BALB/c female mice were immunized intranasally with PBS, OVA (5 μg/mouse) alone, OVA (5 μg/mouse) plus DOTAP/DC-chol liposomes (0.4 μmol/mouse), anionic PS liposomes (0.4 μmol/mouse), or neutral PC liposomes (0.4 μmol/mouse) on days 0 and 7. Serum and nasal washes were collected on day 14. The anti-OVA IgG, IgG1, and IgG2a levels in serum and anti-OVA IgA level in nasal washes were detected by ELISA assay. The data are obtained from at least three independent experiments. The box-plot shows the median value with the 25th-75th percentiles and the error bars indicate the 5th-95th percentiles. Significance was assessed using the Kruskal–Wallis with Dunn’s post–hoc test: *p<0.01.

Since the cationic charge of DOTAP/DC-chol liposomes seem to play a key role on mucosal adjuvant activity, antigen-specific systemic IgG and mucosal IgA responses were examined using anionic PS liposomes and neutral PC liposomes. The results showed that only cationic liposomes induced antigen-specific antibody production, while anionic and neutral liposomes did not have any adjuvant effects, demonstrating the importance of liposome surface charge (Fig 7).

### Antigen-specific IFN-γ and IL–4 production by splenocytes from vaccinated mice *in vitro*

**Fig 8:**
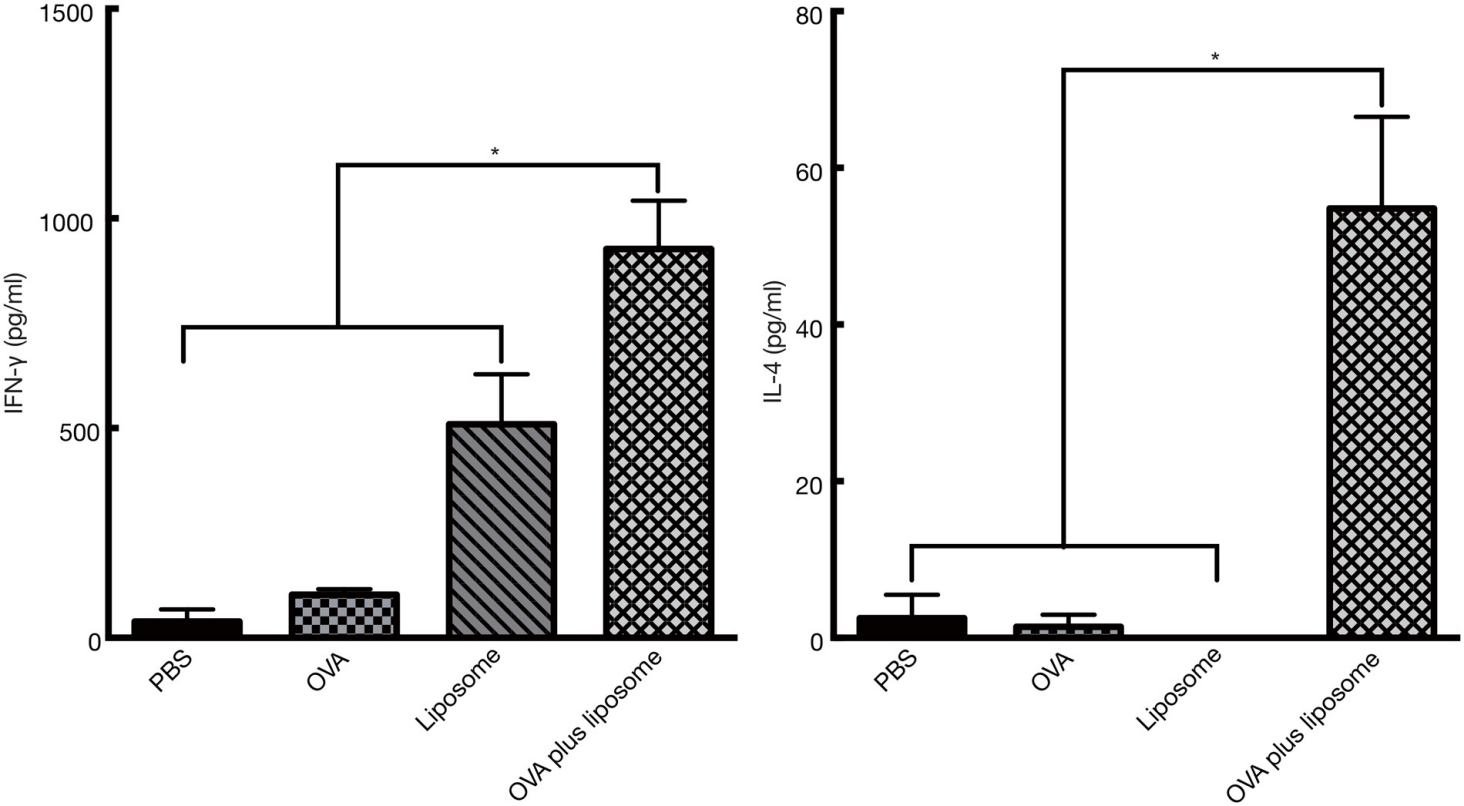
*In vitro* antigen-specific production of IFN-γ and IL–4 in splenocytes and nasal passages from BALB/c mice immunized intranasally with OVA and DOTAP/DC-chol liposomes. Splenocytes from vaccinated BALB/c mice were cultured for 72 h in the presence of OVA (0, 1, 10, or 100 μg/ml). After culture, the supernatants were collected, and concentrations of IFN-γ and IL–4 in the culture supernatants were determined by ELISA assay. The data are representative of at least three independent experiments and are expressed as the mean ± standard deviation for samples assayed in triplicate. Significance was assessed with the *t*-test with Welch correction: *p<0.05.

Vaccination with DOTAP/DC-chol preferentially induced IgG1 production in serum, suggesting that intranasal immunization with cationic liposomes may enhance Th2 immune responses (Fig 1). To characterize the type of immune response elicited by cationic liposomes, we examined IFN-γ and IL–4 secretion by splenocytes re-stimulated with OVA *in vitro*. Compared with splenocytes from mice vaccinated with either PBS, OVA alone, or liposome alone, splenocytes from OVA plus DOTAP/DC-chol liposome-vaccinated mice induced high levels of IL–4 production, while low IFN-γ levels were detected (Fig 8). These results along with the serum IgG1 results indicate that DOTAP/DC-chol liposomes can elicit immune responses utilizing antigen-specific Th2 activity.

### Gene expression profiles of cytokines related to Th1/Th2 immune responses in nasal tissues from vaccinated mice

**Fig 9:**
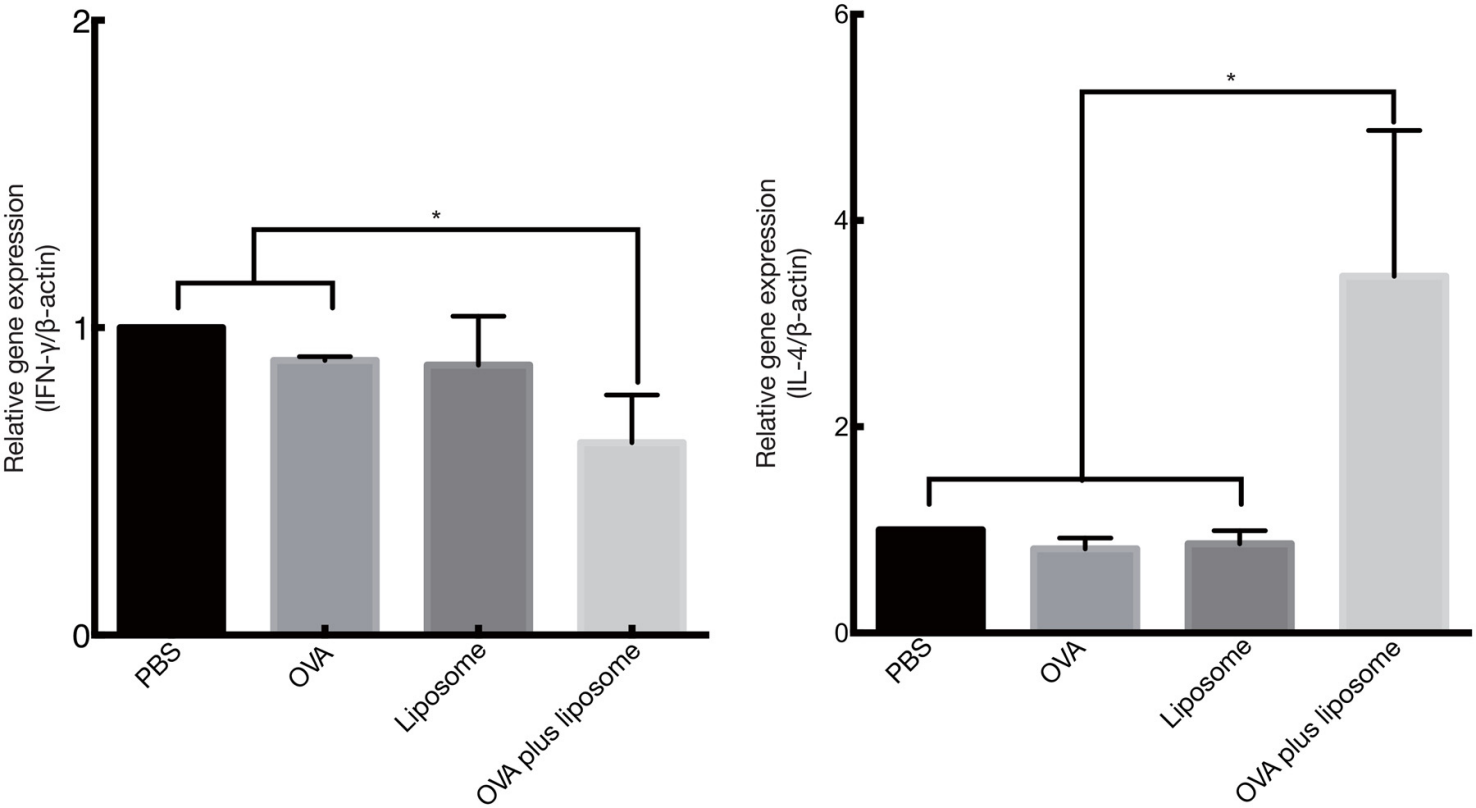
mRNA levels of Th1/Th2 cytokines in vaccinated mice. One hour after the last immunization, spleen and nasal tissues were collected and the expression of mRNAs was quantified by semi-quantitative RT-PCR. Densitometry values for mRNA were normalized to β-actin as an internal standard. The data were representative of at least three independent experiments and are expressed as the mean ± standard deviation for samples assayed in triplicate. Significant difference was assessed using *t*-test with Welch correction: *p<0.05.

The immune response elicited by mucosal vaccination with cationic liposomes was further characterized by assessing the expression of cytokines involved with Th1/Th2 responses. The results showed that vaccination with OVA and DOTAP/DC-chol liposomes induced the expression of IL–4, a Th2 related cytokine, in nasal tissue samples (Fig 9). These results demonstrate that DOTAP/DC-chol liposomes exert a Th2 immune response when administered as a mucosal adjuvant.

### Cationic liposomes enhance OVA-uptake into dendritic cells in NALT

**Fig 10:**
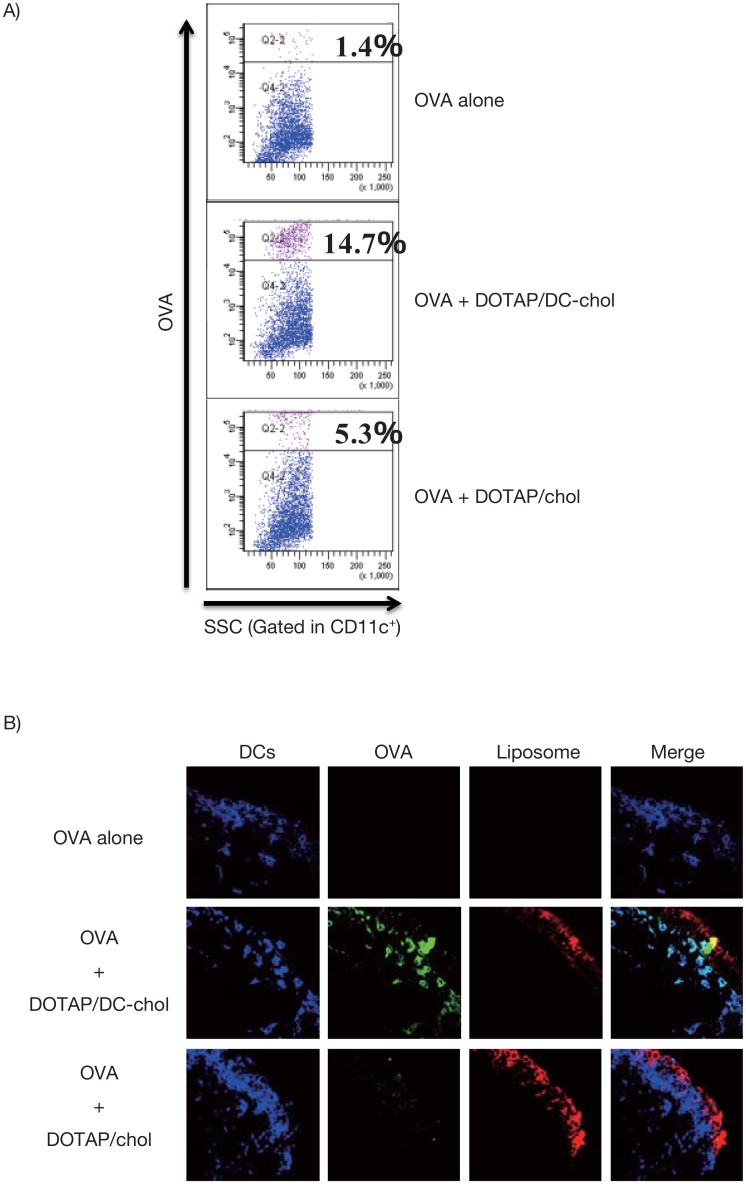
Antigen uptake by CD11c^+^ DCs in NALTs following intranasal administration of Alexa Fluor-labelled OVA plus the cationic liposomes. BALB/c female mice were intranasally administered Alexa Fluor 488-OVA (5 μg/mouse) alone, or Alexa Fluor 488-OVA (5 μg/mouse) plus DOTAP/DC-chol liposomes, or DOTAP/chol liposomes. NALTs were collected 1 h after administration. (A) The NALT cells obtained were stained with anti-mouse CD11c mAb. Alexa Fluor 488-OVA uptake by DCs (based on CD11c^+^ gating) was analysed by flow cytometry, and (B) 8-μm frozen sections of NALTs were stained with antibodies against CD11c (DCs are depicted in blue). Co-localization of OVA, DOTAP/DC-chol liposomes or DOTAP/chol liposomes, and DCs in the cryosections was observed by fluorescence microscopy.

Because effective delivery of antigens into APCs is crucial for the induction of a mucosal immune response [48], we examined whether the cationic liposomes promote antigen uptake into DCs of NALTs as a possible mechanism supporting the adjuvant effects observed. Fig 10 shows that Alexa Fluor 488^+^ cells (OVA^+^ cells) in the DC population were more in NALTs obtained from mice administered Alexa Fluor 488-OVA plus DOTAP/DC-chol liposomes intranasally than in mice administered Alexa Fluor 488-OVA alone. Additionally, we found that DOTAP/DC-chol liposomes more effectively enhanced antigen uptake compared to DOTAP/chol liposomes, implying that this may be a possible reason for the strong mucosal adjuvant effect of DOTAP/DC-chol liposomes.

## Discussion

Vaccination is one of the most powerful tools in the prevention and/or treatment of fatal infectious diseases. In this context, the induction of both mucosal and systemic immune responses would be essential to block the initial infection and prevent further invasion of infections. Therefore, the development of safe and effective mucosal adjuvants is a major goal in vaccine therapy. In the present study, we demonstrate that intranasal administration of OVA with cationic liposomes composed of DOTAP and DC-cholesterol induced antigen-specific mucosal and systemic antibody responses, indicating that this cationic liposome is a potent and safe mucosal adjuvant in mice. The mucosal adjuvant activity of DOTAP/DC-chol liposomes shown here may provide a novel mucosal vaccine therapy approach for the prevention and treatment of various infectious diseases, including fungal and bacterial pathogens.

We demonstrate for the first time that intranasal vaccination with cationic liposomes elicits antigen-specific antibody production in nasal mucosal tissue and serum in a dose-dependent manner (Fig 2), as well as enhances the kinetics of antibody expression in serum and mucosal tissues (Figs 1 and 3). This liposomal activity was dependent on surface charge (Fig 7), but independent of particle size (Fig 4). There are many reports in the literature of the potential toxicity associated with cationic liposomes [46, 47]. However, we have successfully demonstrated the safety of the DOTAP/DC-chol liposomes used in this study. In particularly, they did not show any toxicity *in vivo*, having no effect on body weight (Fig 6A) or the induction of an inflammatory cytokine in mice (Fig 6B).

While the precise molecular mechanisms of mucosal adjuvant effects induced by cationic liposomes are not known, it is clear that a cationic charge is required for this activity. Generally, adjuvants employ the following mechanisms to elicit their adjuvant effects: (1) sustained release of antigen at the site of injection, which is referred to as the ‘depot effect’, (2) increased antigen uptake and presentation to APCs, including DCs, and (3) activation of the innate immune system [48]. Here we demonstrated that DOTAP/DC-chol liposomes promote antigen uptake into the DCs of NALTs (Fig 10).

Recent studies have shown that cationic molecules, such as polyethyleneimine (PEI) [19] and a nanoparticle composed of poly-γ-glutamic acid (γ-PGA) and chitosan [11], have mucosal adjuvant effects. Other possible modes of action for adjuvants are through activation of innate immune responses that serve as a bridge to adaptive immunity. A recent study reported that alum, an aluminium adjuvant, causes cell death and the release of cellular DNA, which acts as an immunostimulatory signal mediating alum adjuvant activity [49]. The adjuvant activity of PEI requires the release of host double-stranded DNA (dsDNA) to trigger an innate immune response [19]. Previously, we found that cationic liposomes can induce mitochondrial oxidative stress-mediated cell death of macrophages *in vitro* [36, 50–53]. Cationic liposomes may elicit a danger signal by *in vivo* release of damage-associated molecular pattern molecules (DAMPs) such as dsDNA, uric acid crystals, adenosine triphosphate (ATP), and high mobility group box–1 (HMGB1) protein [18, 54], which triggers an innate immune response that can induce a humoral immune response. This possibility will need to be clarified in future experiments.

We further characterized the type of immune response triggered by cationic liposomes by assessing the production of cytokines and serum IgG subclass responses. Cationic liposomes preferentially induced Th2 responses based on the following study observations: (1) DOTAP/DC-chol preferentially induced IgG1 production in serum (Fig 1), and (2) NALTs and splenocytes from OVA plus DOTAP/DC-chol liposome treated mice had increased IL–4 expression (Figs 8 and 9). The mucosal adjuvant activity of the different cationic liposomes varied depending on the lipid formulation. The combination of DOTAP and DC-cholesterol was most active in this study (Fig 1). However, it may be possible to develop even more potent and safe cationic liposomes by evaluating other lipids, and we are currently investigating this possibility.

## Conclusions

In this study, intranasal vaccination with cationic liposomes and protein antigen elicited antigen-specific antibody responses in mucosal tissues and in systemic circulation. While the underlying mechanisms of the mucosal adjuvant effect that were observed are not yet understood, these findings demonstrate that this approach can be used for the development of mucosal vaccines to combat infectious diseases caused by pathogenic microbes. Studies are in progress for developing a mucosal vaccine for bacterial and fungal infections by using cationic liposomes as a mucosal adjuvant.

## Supporting Information

S1 Fig Change in particle size of the cationic liposomes. (EPS)
